# Multiplex Human Malaria Array: Quantifying Antigens for Malaria Rapid Diagnostics

**DOI:** 10.4269/ajtmh.19-0763

**Published:** 2020-03-16

**Authors:** Ihn Kyung Jang, Abby Tyler, Chris Lyman, John C. Rek, Emmanuel Arinaitwe, Harriet Adrama, Maxwell Murphy, Mallika Imwong, Stephane Proux, Warat Haohankhunnatham, Rebecca Barney, Andrew Rashid, Michael Kalnoky, Maria Kahn, Allison Golden, François Nosten, Bryan Greenhouse, Dionicia Gamboa, Gonzalo J. Domingo

**Affiliations:** 1 Diagnostics, PATH, Seattle, Washington;; 2 Quansys Biosciences, Logan, Utah;; 3 Infectious Diseases Research Collaboration, Kampala, Uganda;; 4 Department of Medicine, University of California at San Francisco, San Francisco, California;; 5 Faculty of Tropical Medicine, Department of Molecular Tropical Medicine and Genetics, Mahidol University, Bangkok, Thailand;; 6 Faculty of Tropical Medicine, Mahidol-Oxford Tropical Medicine Research Unit, Shoklo Malaria Research Unit, Mahidol University, Mae Sot, Thailand;; 7 Nuffield Department of Medicine, Centre for Tropical Medicine and Global Health, University of Oxford, Oxford, United Kingdom;; 8 Department of Cellular and Molecular Sciences, Institute of Tropical Medicine Alexander von Humboldt, Universidad Peruana Cayetano Heredia, Lima, Peru

## Abstract

Malaria antigen detection through rapid diagnostic tests (RDTs) is widely used to diagnose malaria and estimate prevalence. To support more sensitive next-generation RDT development and screen asymptomatic malaria, we developed and evaluated the Q-Plex^™^ Human Malaria Array (Quansys Biosciences, Logan, UT), which quantifies the antigens commonly used in RDTs—*Plasmodium falciparum*–specific histidine-rich protein 2 (HRP2), *P. falciparum-*specific lactate dehydrogenase (*Pf* LDH), *Plasmodium vivax–*specific LDH (*Pv* LDH), and Pan malaria lactate dehydrogenase (Pan LDH), and human C-reactive protein (CRP), a biomarker of severity in malaria. At threshold levels yielding 99.5% or more diagnostic specificity, diagnostic sensitivities against polymerase chain reaction–confirmed malaria for HRP2, *Pf* LDH, *Pv* LDH, and Pan LDH were 92.7%, 71.5%, 46.1%, and 83.8%, respectively. *P. falciparum* culture strains and samples from Peru indicated that HRP2 and *Pf* LDH combined improves detection of *P. falciparum* parasites with *hrp2* and *hrp3* deletions. This array can be used for antigen-based malaria screening and detecting *hrp2/3* deletion mutants of *P. falciparum*.

## INTRODUCTION

There are more than 200 million cases of human malaria worldwide, with some countries in the control phase of malaria and others seeking to eliminate malaria.^1^ Malaria diagnosis and treatment is mostly through detection of the parasite by microscopy or parasite antigens through rapid diagnostic tests (RDTs).^2^ Many commonly used RDTs target two malaria antigens: histidine-rich protein (HRP2) and the essential enzyme, *Plasmodium* LDH (pLDH).^3^ Histidine-rich protein 2, a *Plasmodium falciparum*–specific protein, and its homolog HRP3 are used as biomarkers to confirm *P. falciparum* infection. Unfortunately, RDTs recognizing the common epitopes found in HRP2 and HRP3 result in failed diagnosis for clinical *P. falciparum* infections because of partial or complete deletions of *hrp2* and *hrp3*, and consequently no expression of proteins.^4^ Characterization of *hrp2/3* deletions by polymerase chain reaction (PCR) is time consuming and expensive for larger scale clinical studies.^4^ Antibodies targeting a conserved epitope in pLDH can detect all human *Plasmodium* species Pan malaria lactate dehydrogenase (Pan LDH). pLDH can also be used for species-specific detection for *P. falciparum* and *P. vivax* infection using antibodies that recognize species-specific epitopes in *P. falciparum-*specific lactate dehydrogenase (*Pf* LDH) and *P. vivax–*specific LDH (*Pv* LDH).^5,6^

The performance of RDTs is driven by the underlying relationship between antigen concentration and parasite density. This relationship is defined by multiple factors including antigen turnover and decay rates, affinity characteristics of the antigen capture reagents, whether infections are recent or asymptomatic, and probably also by host factors such as previous exposure to malaria.^7–10^ Two quantitative multiplex tests, bead-based assay and chemiluminescence-based ELISA, have been described.^11,12^ The bead-based assay detects Pan aldolase, Pan LDH, and HRP2, whereas the ELISA detects HRP2, *Pv* LDH, Pan LDH, and C-reactive protein (CRP). An expanded version of this ELISA including an assay for *Pf* LDH was developed for research use and is now commercially available as the Q-Plex^™^ Human Malaria Array (5-Plex [Quansys Bioscience, Logan, UT]). In this study, the diagnostic performance of this new test was assessed.

## METHODS

### Ethics and source of specimen.

Clinical samples from asymptomatic infections in Myanmar and Uganda were collected under informed consent and with institutional review board (IRB) approvals, as described previously.^13^ A randomly selected subset of samples was used in this study. Seventy-five clinical samples with *P. falciparum* or *P. vivax* infections from symptomatic infections in Vietnam, which were also collected under informed consent and IRB approval, were purchased from Discovery Life Sciences (Los Osos, CA). Twenty-six *P. falciparum* clinical samples were obtained with informed consent and with IRB approval from Universidad Peruana Cayetano Heredia (UPCH, Lima, Peru) (UPCH 52707). *Plasmodium malariae–* and *Plasmodium ovale*–infected blood samples were obtained from the Foundation for Innovative New Diagnostics (FIND, Geneva, Switzerland) Specimen Bank.

Five *P. falciparum* laboratory strains were used in this study, W2, D10, Dd2, and HB3 (BEI Resources, Manassas, VA), and 3BD5 (the National Institute of Allergy and Infectious Diseases, Bethesda, MD). The wild-type strain W2 is *hrp2*^*+*^/*hrp3*^*+*^, D10 and Dd2 are *hrp2*^*−*^*/hrp3*^*+*^, HB3 is *hrp2*^*+*^*/hrp3*^*−*^, and 3BD5 is *hrp2*^*−*^/*hrp3*^*−*^. All strains were cultured and harvested at the ring stage (> 99%) after synchronization, as described previously.^12^ The parasite number was determined by microscopy. Parasite-infected red blood cell pellets were used to generate a dilution series derived from a pool of uninfected blood (see Supplemental Methods).

### Laboratory tests for HRP2/pLDH and parasite DNA.

Quantification of malaria antigens and CRP was performed using the 5-Plex (Quansys Bioscience) (see Supplemental Methods). The speciation of all field samples except those from Vietnam was confirmed by quantitative PCR methods.^13^ Photo-induced electron transfer real-time PCR was used to characterize samples from Vietnam (see Supplemental Methods). The samples from Peru were previously characterized for the presence or absence of *hrp2* and *hrp3* by PCR and confirmed for HRP proteins by conventional ELISA.^14^

### Statistical analysis.

All statistical analyses were performed using GraphPad Prism, version 6.0 (GraphPad, San Diego, CA). The detail in data analysis is described in Supplemental Methods.

## RESULTS

### Performance of 5-plex array.

**Figure 1.**
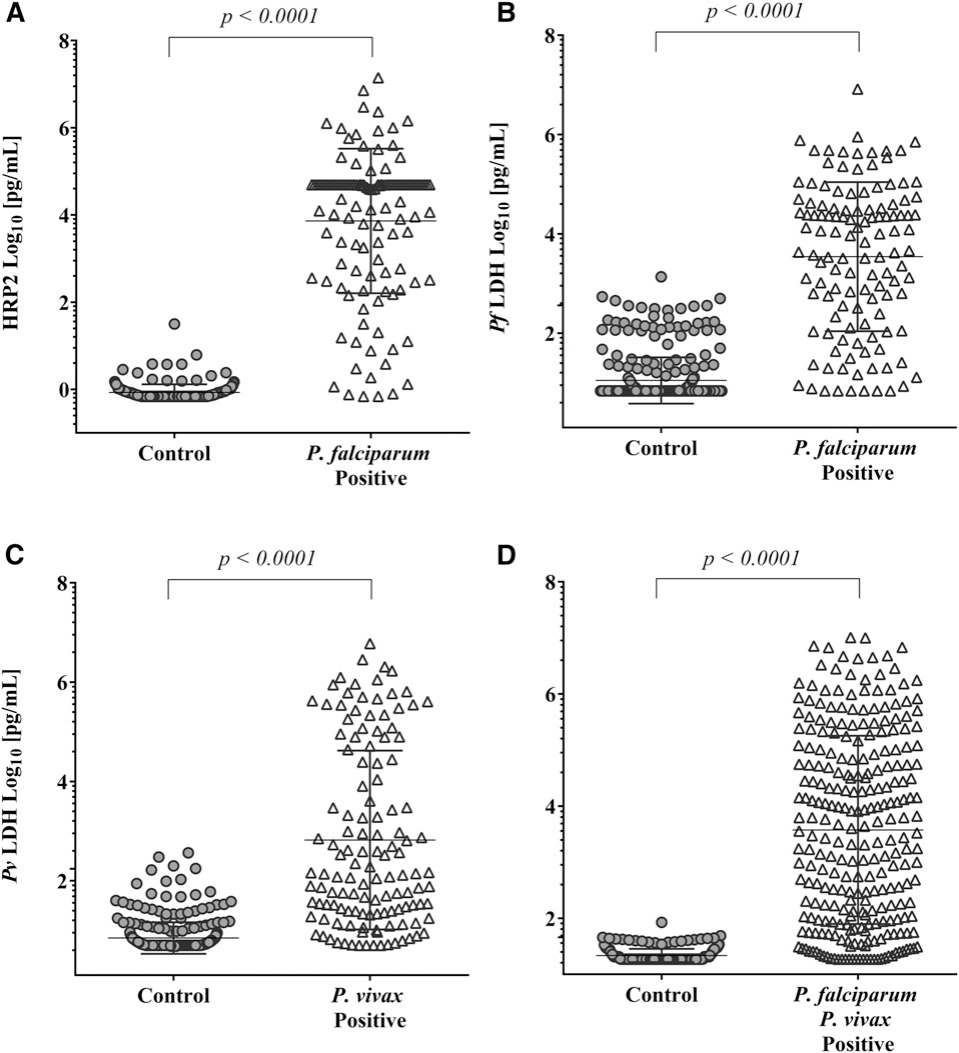
Dot plots of the concentration for each biomarker as determined on the 5-Plex infection-negative and infection-positive samples (as summarized in Supplemental Table 2). (**A**) Histidine-rich protein 2 (HRP2), (**B**) *Plasmodium falciparum-*specific lactate dehydrogenase (*Pf* LDH), (**C**) *P. vivax–*specific LDH (*Pv* LDH), and (**D**) Pan malaria lactate dehydrogenase (Pan LDH) assays. Malaria-negative samples (no parasites, *n* = 196) from Uganda and Vietnam were used as controls for the HRP2 and Pan LDH assays, whereas samples that do not have the corresponding *Plasmodium* species (non–*P. falciparum* malaria, *n* = 128; non–*P. vivax* malaria, *n* = 123) were used as controls for *Pv* LDH and *Pf* LDH assays to address the false-positive results from cross-reactivity of pLDH. The longer horizontal line in each blot indicates the mean, and the upper and lower shorter lines indicate one standard deviation (SD). The Mann–Whitney U-test was used to calculate *P*-values; *P* < 0.05 was considered statistically significant.

The analytical performance of the 5-Plex was evaluated based on antigen quantification as determined from the pixel intensity on the array spot relative to a standard curve (Supplemental Table 1 and Supplemental Table 2). The 5-Plex was challenged with a validation sample set consisting of 462 blood samples from PCR-confirmed positive and negative cases with *P. falciparum* and *P. vivax* infection from Uganda, Myanmar, and Vietnam. All four malaria biomarkers, HRP2, Pan LDH, *Pf* LDH, and *Pv* LDH, found in significantly higher amounts in the respective malaria-positive samples are compared with the negative control group (*P* < 0.0001) (Figure 1, Supplemental Table 3). Receiver operating characteristic (ROC) analysis was conducted, and the area under the ROC curves (AUCs) with 95% CIs for each biomarker was calculated in relation to the discrimination between malaria cases and controls. The AUCs were 0.979 (95% CI: 0.960–0.998) for HRP2, 0.939 (95% CI: 0.911–0.968) for *Pf* LDH, 0.926 (95% CI: 0.898–0.954) for *Pv* LDH, and 0.948 (95% CI: 0.928–0.968) for Pan LDH (Supplemental Figure 1).

Optimal cutoff points were calculated targeting a specificity of ≥ 99.5% (Table 1) against PCR-confirmed negative samples or non-cognate *Plasmodium* subspecies infection. From analyzing the validation sample set described in Figure 1, the HRP2 assay displayed the best diagnostic performance characteristics with a sensitivity and specificity of 92.7% and 99.5%, respectively. Analysis with 15 validation samples with dual infection of *P. falciparum* and *P. vivax* revealed that the 5-Plex identified seven samples as coinfected, with the remaining eight samples being only *P. vivax* positive because of very low *P. falciparum* parasitemia in the range of 1–40 p/μL.

**Table 1:** Performance characteristics for each malaria antigen assay used in the 5-Plex HRP2, *Pf* LDH for *P. falciparum*, *Pv* LDH for *P. vivax*, and Pan LDH for all malaria species

Analyte	Target polymerase chain reaction-confirmed infection	Threshold (pg/mL)	Sensitivity (95% CI)	Specificity (95% CI)
HRP2	*P. falciparum*	6.9	92.7 (86.6–96.6)	99.5 (97.2–100)
*Pf* LDH	*P. falciparum*	553.6	71.5 (62.7–79.3)	99.7 (98.3–100)
*Pv* LDH	*P. vivax*	314.8	46.1 (37.3–55.1)	99.7 (98.3–100)
Pan LDH	All malaria	49.0	83.8 (78.9–88.1)	99.5 (97.2–100.0)

HRP2 = histidine-rich protein 2; *P. falciparum* = *Plasmodium falciparum*; *Pf* LDH = *P. falciparum-*specific lactate dehydrogenase; *Pv* LDH = *P. vivax–*specific LDH; Pan LDH = Pan malaria lactate dehydrogenase.

A dilution series of five *P. falciparum* strains in the range of 66.7–0.3 p/μL with variable *hrp2* and *hrp3* genotypes were also analyzed on the 5-Plex. The HRP2 assay detected parasites from two strains, the wild-type W2 *(hrp2*^*+*^*/hrp3*^*+*^) and the HB3 (*hrp2*^*+*^/*hrp3*^*−*^), to as few as 0.8 p/μL and 0.3 p/μL, respectively. In parallel, the *Pf* LDH assay detected parasites at 66.7 p/μL and 22.2 p/μL, respectively (Supplemental Figure 2). Screening of *hrp* mutants with the *Pf* LDH assay using *hrp2* deletion strains D10 and Dd2 and 3BD5, a *hrp2/3* double mutant detected parasitemia of 7.4 p/μL, 22.2 p/μL, and 66.7 p/μL, respectively.

The 5-Plex was also evaluated with a panel of 26 parasite count–adjusted samples derived from clinical samples collected in Peru and previously characterized for *hrp2/3* deletions by PCR.^14^ All 26 samples were accurately identified as *P. falciparum* by the 5-Plex by detecting either HRP2 or *Pf* LDH. A subset of nine from 12 (75%) samples with *hrp2/3* deletion in this panel was correctly identified as HRP negative with the 5-Plex HRP2 assay (Table 2).

**Table 2:** Performance of the 5-Plex to identify *P. falciparum* using *Pf* LDH and Pan LDH assays with *hrp2/3*–deleted *P. falciparum* samples

Category	*hrp*2*/3* deletion by polymerase chain reaction	5-Plex, positive sample number.				% Accuracy	
	Sample number	HRP2	*Pf* LDH	*Pv* LDH	Pan LDH	*Pf c*lassification	hrp2/3 deletion
hrp2+/hrp3+	5	5	5	0	5	100	NA
hrp2+/hrp3−	6	6	6	0	6	100	NA
hrp2−/hrp3+	3	1*	3	0	3	100	NA
hrp2−/hrp3−	12	3†	12	0	12	100	75.0

HRP = histidine-rich protein; NA = not applicable; *P. falciparum* = *Plasmodium falciparum; Pf* LDH = *P. falciparum-*specific lactate dehydrogenase; Pan LDH = Pan malaria lactate dehydrogenase; *Pv* LDH = *P. vivax–*specific LDH.

* 230 ng/mL of HRP2 by the 5-Plex; no conventional ELISA data available.

† One sample was detected for 40,000 pg/mL of HRP2 by the 5-Plex but was not quantifiable by the conventional ELISA. Two other samples were 18.9 and 9 pg/mL of HRP2 by the 5-Plex; no conventional ELISA data available.

### Cross-reactivity with other *Plasmodium* species.

The 5-Plex was further investigated with 17 *P. malariae* and eight *P. ovale* PCR-confirmed samples. Of the 17 *P. malariae*–positive samples, 14/17 (82.4%) were positive by the Pan LDH assay and only 5/17 (29.4%) were positive by the *Pf* LDH assay (Supplemental Figure 3**)**. The *P. malariae* samples had relatively high parasite densities in the range of 75.2–988.3 p/μL. Of the eight PCR *P. ovale*–positive samples, 3/8 (37.5%) were positive by Pan LDH assay, with parasite densities in the range of 0.1–8.0 p/μL. None (0/8) was positive by *Pf* LDH assay. The *P. malariae* and *P. ovale* samples were negative with the HRP2 and *Pv* LDH assays.

## DISCUSSION

The 5-Plex is able to differentiate *P. falciparum* infections, including those with *hrp2/3* deletions, *P. vivax* infection, and also detect *P. ovale* and *P. malariae*. Similar multiplex assays have been developed on the Luminex bead-based platform but are not available as commercial kits.^11,15,16^

In contrast to the previously described 4-Plex array,^12^ the performance of the new 5-Plex is significantly improved: the prozone effect in the HRP2 assay is mitigated along with the wider dynamic range (Supplemental Figure 4),^17^ and through the *Pf* LDH, it can identify samples with suspected *hrp2/3* deletions.

At a specificity of > 99.5%, the sensitivity for detecting any species of malaria using the Pan LDH assay was 83.8% and for *P. falciparum* infection using HRP2 was 92.7%. When identifying *P. falciparum* and *P. vivax* infections through the *Pf* LDH and *Pv* LDH assays, the sensitivity was reduced to 71.5% and 46.1%, respectively. The *Pf* LDH assay does show cross-reactivity with *P. malariae*. The sequence similarity of the related proteins from *P. malariae* and *P. falciparum* may contribute to cross-reactivity. In addition, elevated levels of CRP, an acute phase inflammatory protein, were consistently observed in febrile malaria-positive individuals, in contrast to asymptomatic malaria-positive individuals (Supplemental Figure 5), supporting the correlation of the CRP level with complications in malaria.^18,19^

The data presented in this study suggest that the Q-Plex^™^ Human Malaria Array can be used on whole blood samples to operate high throughput screening tool for malaria surveillance including the identification of *hrp2/3*–deleted *P. falciparum* before resorting to more complex molecular tests for the gene deletions.^4^ Although HRP2 persists after malaria clearance, the pLDH level rapidly declines on treatment.^20^ The capability of the array to simultaneously quantify these malaria antigens may be of significant value to the future studies that could provide deep insight into the malaria infection status classification as well as the speciation of suspected malaria.

## Supplemental file

Supplemental materials

## References

[1] WHO, 2018 World Malaria Report 2018. Geneva, Switzerland: World Health Organization.

[2] Wongsrichanalai C Barcus MJ Muth S Sutamihardja A Wernsdorfer WH, 2007 A review of malaria diagnostic tools: microscopy and rapid diagnostic test (RDT). Am J Trop Med Hyg 77: 119–127. 18165483

[3] Mouatcho JC Goldring JP, 2013 Malaria rapid diagnostic tests: challenges and prospects. J Med Microbiol 62: 1491–1505. 24048274 10.1099/jmm.0.052506-0

[4] Cheng Q Gatton ML Barnwell J Chiodini P McCarthy J Bell D Cunningham J, 2014 *Plasmodium falciparum* parasites lacking histidine-rich protein 2 and 3: a review and recommendations for accurate reporting. Malar J 13: 283. 25052298 10.1186/1475-2875-13-283 PMC4115471

[5] Hurdayal R Achilonu I Choveaux D Coetzer TH Dean Goldring JP, 2010 Anti-peptide antibodies differentiate between plasmodial lactate dehydrogenases. Peptides 31: 525–532. 20093160 10.1016/j.peptides.2010.01.002

[6] Piper RC Buchanan I Choi YH Makler MT, 2011 Opportunities for improving pLDH-based malaria diagnostic tests. Malar J 10: 213. 21806821 10.1186/1475-2875-10-213 PMC3163226

[7] Ho MF 2014 Circulating antibodies against *Plasmodium falciparum* histidine-rich proteins 2 interfere with antigen detection by rapid diagnostic tests. Malar J 13: 480. 25481825 10.1186/1475-2875-13-480 PMC4295572

[8] Markwalter CF Jang IK Burton RA Domingo GJ Wright DW, 2017 Biolayer interferometry predicts ELISA performance of monoclonal antibody pairs for *Plasmodium falciparum* histidine-rich protein 2. Anal Biochem 534: 10–13. 28698001 10.1016/j.ab.2017.07.010 PMC5552614

[9] Dalrymple U Arambepola R Gething PW Cameron E, 2018 How long do rapid diagnostic tests remain positive after anti-malarial treatment? Malar J 17: 228. 29884184 10.1186/s12936-018-2371-9 PMC5994115

[10] Ranadive N 2017 Limitations of rapid diagnostic testing in patients with suspected malaria: a diagnostic accuracy evaluation from Swaziland, a low-endemicity country aiming for malaria elimination. Clin Infect Dis 64: 1221–1227. 28369268 10.1093/cid/cix131 PMC5399938

[11] Rogier E 2017 Bead-based immunoassay allows sub-picogram detection of histidine-rich protein 2 from *Plasmodium falciparum* and estimates reliability of malaria rapid diagnostic tests. PLoS One 12: e0172139. 28192523 10.1371/journal.pone.0172139 PMC5305216

[12] Jang IK 2019 Simultaneous quantification of *Plasmodium* antigens and host factor C-reactive protein in asymptomatic individuals with confirmed malaria by use of a novel multiplex immunoassay. J Clin Microbiol 57: e00948-18. 30404944 10.1128/JCM.00948-18 PMC6322473

[13] Das S 2017 Performance of a high-sensitivity rapid diagnostic test for *Plasmodium falciparum* malaria in asymptomatic individuals from Uganda and Myanmar and naive human challenge infections. Am J Trop Med Hyg 97: 1540–1550. 28820709 10.4269/ajtmh.17-0245 PMC5817764

[14] Gamboa D 2010 A large proportion of *P. falciparum* isolates in the Amazon region of Peru lack pfhrp2 and pfhrp3: implications for malaria rapid diagnostic tests. PLoS One 5: e8091. 20111602 10.1371/journal.pone.0008091 PMC2810332

[15] Plucinski MM 2019 Screening for pfhrp2/3-deleted *Plasmodium falciparum*, non-falciparum, and low-density malaria infections by a multiplex antigen assay. J Infect Dis 219: 437–447. 30202972 10.1093/infdis/jiy525 PMC6325347

[16] Plucinski MM Rogier E Dimbu PR Fortes F Halsey ES Aidoo M, 2017 Estimating the added utility of highly sensitive histidine-rich protein 2 detection in outpatient clinics in sub-saharan africa. Am J Trop Med Hyg 97: 1159–1162. 28722629 10.4269/ajtmh.17-0262 PMC5637620

[17] Luchavez J Baker J Alcantara S Belizario V Jr. Cheng Q McCarthy JS Bell D, 2011 Laboratory demonstration of a prozone-like effect in HRP2-detecting malaria rapid diagnostic tests: implications for clinical management. Malar J 10: 286. 21957869 10.1186/1475-2875-10-286 PMC3214175

[18] Gillespie SH Dow C Raynes JG Behrens RH Chiodini PL McAdam KP, 1991 Measurement of acute phase proteins for assessing severity of *Plasmodium falciparum* malaria. J Clin Pathol 44: 228–231. 1707416 10.1136/jcp.44.3.228 PMC496944

[19] Andrade BB Reis-Filho A Souza-Neto SM Clarencio J Camargo LM Barral A Barral-Netto M, 2010 Severe *Plasmodium vivax* malaria exhibits marked inflammatory imbalance. Malar J 9: 13. 20070895 10.1186/1475-2875-9-13 PMC2837053

[20] Iqbal J Siddique A Jameel M Hira PR, 2004 Persistent histidine-rich protein 2, parasite lactate dehydrogenase, and panmalarial antigen reactivity after clearance of *Plasmodium falciparum* monoinfection. J Clin Microbiol 42: 4237–4241. 15365017 10.1128/JCM.42.9.4237-4241.2004 PMC516301

