# Supplementary material for: Multiplex Human Malaria Array: Quantifying Antigens for Malaria Rapid Diagnostics
Source: Am J Trop Med Hyg. 2020 Mar 16;102(6):1366–9. doi: 10.4269/ajtmh.19-0763 (PMC7253106; doi:10.4269/ajtmh.19-0763)
Supplement: Supplementary file 1 [file tpmd190763.SD1.docx]

**Supplemental Materials**

**Supplemental Methods**

***P. falciparum* culture**

*P. falciparum* laboratory adapted strains, W2, HB3, Dd2, D10, and 3BD5, were cultured in leukocyte-depleted human O^+^ erythrocytes diluted at 4% hematocrit in RPMI-1640 media supplemented with HEPES (Gibco), sodium bicarbonate (Sigma), hypoxanthine (Sigma), gentamycine (Sigma), and either 10% human serum (Interstate Blood Bank) or 0.5% AlbuMAX II (Invitrogen) using a modified *in vitro* culture technique. Parasite cultures were synchronized with 5% D-sorbitol and harvested at ring stage (> 99 %). Parasitemia of culture was determined by microscopic analysis. A threefold serial dilution from 66.7 p/μL to 0.3 p/μL was then made in whole blood (Interstate Blood Bank).

**Procedure for the 5-Plex**

All de-identified human clinical samples and cell culture samples were subjected to blinded experiments by two experienced operators to perform the 5-Plex. The manufacturer’s instruction manual can be found on the following link (<http://quansysbio.com/wp-content/uploads/2020/01/Malaria-Chemi-5-Plex-Manual-2-8-05-19-lr.pdf>). Briefly, calibrators and samples were prepared according to the manufacturer’s protocol. Each sample was tested both neat and diluted 20-fold unless noted otherwise. After addition of 50 µL of calibrators and samples, the plate was incubated at room temperature with shaking at 500 revolutions per minute (rpm) for 2 hours. Plates were then washed with proprietary wash buffer using an automated plate washer. A 50 µL aliquot of detection mix containing proprietary ingredients, including biotinylated antibodies and buffer, was added to each well, and the plate was incubated with shaking for another hour and then washed again. For detection, a 50 µL aliquot of horseradish peroxide (HRP)-conjugated streptavidin solution was added to each well and then incubated with shaking for 30 minutes. After a final wash, a 50 µL aliquot of chemiluminescent substrate solution was added to each well and the chemiluminescent intensity from the array spots in each well was immediately measured using the Q-View Imager Pro (Quansys Biosciences) at an exposure time of 300 seconds.

**PET-PCR**

DNA was extracted from 100 μL of whole blood from Discovery Life Sciences (DLS) using the QIAamp DNA Mini Kit (Qiagen Inc, Chatsworth, CA, USA). *Plasmodium* genus-specific PET-PCR was performed in duplicate using 5 μL of DNA as previously described.^1^ For detection of *P. falciparum* and *P.* *vivax*, the reactions were set up in duplicate with species-specific PET-PCR primers which have been verified previously.^1,2^ Positive controls consisting of samples with known *Plasmodium* species and nuclease-free water as a negative control were included in each run.

**Assay validation.**

The theoretical lower limit of detection (LLD) is the lowest concentration of analyte that can be detected with a specific degree of probability. LLD was calculated using the following formula: LLD = 2*(Standard deviation of negative control pixel intensities before Negative Well Subtraction) * Lower limit of quantification (LLOQ)/(Difference between pixel intensity of lowest standard and negative control). The LLOQ and upper limit of quantification (ULOQ) are defined as the lowest calibrator point and the highest calibrator point for which the concentration can be back-calculated on the non-linear regression curve with 80% to 120% accuracy and a coefficient of variation (CV) of less than 30%. The prozone effect on the 4-Plex and 5-Plex was also evaluated as presence of false-low results due to presence of excess amounts of antigens or antibodies in immune-reactions.

**Statistical analysis**

All statistical analysis was performed using GraphPad Prism, version 6.0 (GraphPad Software, California, USA). Characteristics of assay performance through receiver operating characteristic curve (ROC) analysis was performed. For non-parametric data, the differences between two groups were analyzed using the Mann-Whitney U-test. ROC analysis was conducted and the area under the ROC curves (AUC) with 95% confidence intervals for each biomarker was calculated in relation to the discrimination between malaria cases and controls. To determine a threshold level (yielding a specificity of 99.5% or more for each of biomarkers, the sensitivity and the specificity were calculated by the formulas, (true positives)/(true positives + false negatives) and (true negatives)/(true negatives + false positives), respectively. Quantitative PCR was considered as reference assay to estimate the diagnostic sensitivity and diagnostic specificity of assay for each biomarker. One-way analysis of variance (ANOVA) and Tukey’s multiple comparison test was used to determine whether fever symptom had any significant effect on CRP level. For all analyses, *p* values less than 0.05 were considered statistically significant.

**Reference**

1. Lucchi NW, Narayanan J, Karell MA, Xayavong M, Kariuki S, DaSilva AJ, Hill V, Udhayakumar V, 2013. Molecular diagnosis of malaria by photo-induced electron transfer fluorogenic primers: PET-PCR. PLoS One: 8: e56677-10.1371/journal.pone.0056677
2. Kudyba HM, Louzada J, Ljolje D, Kudyba KA, Muralidharan V, Oliveira-Ferreira J, Lucci NW, 2019. Field evaluation of malaria malachite green loop-mediated isothermal amplification in health posts in Roraima state, Brazil. Malar J 18:98

**Supplemental Table 1.** Characterization of calibrator curves generated during assay validation
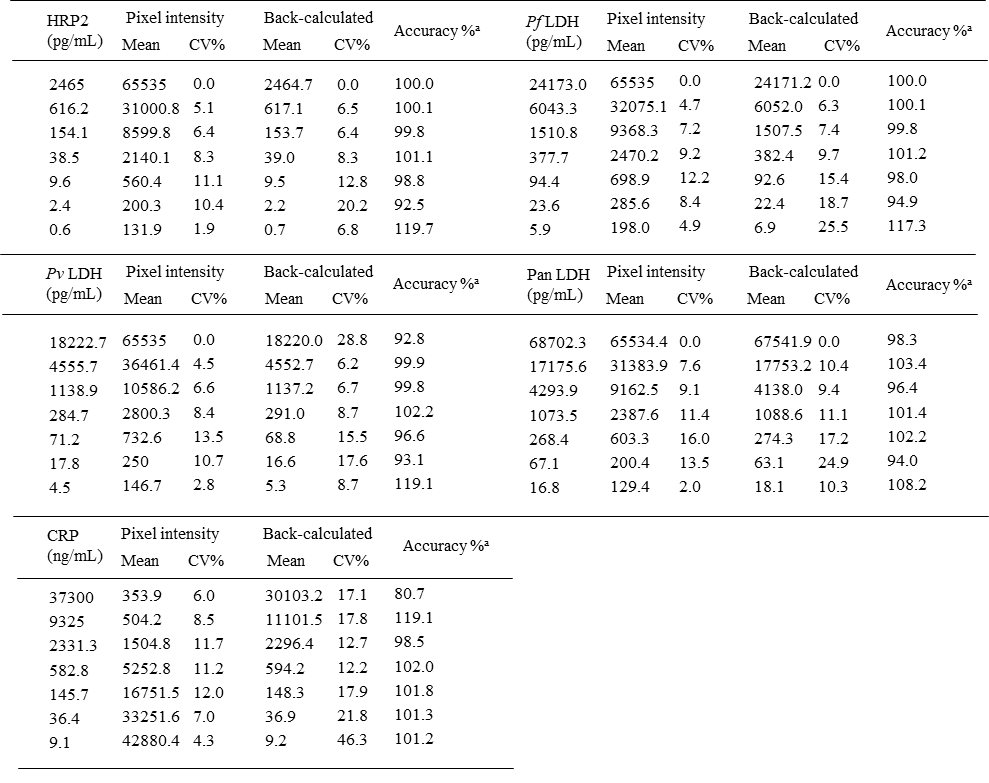


^a^Accuracy was calculated with data collected from 14 analytical runs using the formula; observed concentration/expected concentration) x 100

**Supplemental Table 2.** Analytical performance characteristics of the 5-Plex.

| **Target** | **Unit** | **LLD^a^** | **LLOQ^b^** | **ULOQ^c^** |
| --- | --- | --- | --- | --- |
| HRP2 | pg/mL | 0.6 | 0.7 | 2,465 |
| *Pf* LDH | pg/mL | 4.3 | 6.9 | 24,171 |
| *Pv* LDH | pg/mL | 4.0 | 5.3 | 18,220 |
| Pan LDH | pg/mL | 16.6 | 18.1 | 67,542 |
| CRP | ng/mL | 5.2 | 36.9 | 30,103 |

Abbreviation: LLD, lower limit of detection; LLOQ, lower limit of quantification; ULOQ, upper limit of quantification.

^a^ LLD = 2 * (Standard deviation of negative control pixel intensities) * LLOQ/(Difference between pixel intensity of lowest standard and negative control).

^b^ LLOQ, ULOQ: 80% to 120% accuracy and a coefficient of variation of less than 30%.

^c^ ULOQ established by sample dilution scheme enabling the quantification.

**Supplemental Table 3.** Mean concentration and 95% confidence interval of the 5-Plex in relation to PCR results. Samples from asymptomatic individuals in Uganda and Myanmar study sites were tested at additional 20-fold dilution while samples collected from symptomatic individuals carrying increased number of parasites in Vietnam were tested at further serial dilutions for quantification of biomarkers. The positive and control samples correspond to the dot plots in **Figure 1**.

| **Biomarkers** | **Category** | **Mean (SD)**  **pg/mL** | **95% CI of mean** | ***p* value** |
| --- | --- | --- | --- | --- |
| HRP2 | Control (n = 196) | 1.1 (2.3) | 0.7–1.4 | < 0.0001 |
|  | Positive (n = 123) | 308,357 (1.5e+006) | 48,009–568,704 |  |
| *Pf* LDH | Control (n = 324)^a^ | 34.2 (104.8) | 22.8–45.7 | < 0.0001 |
|  | Positive (n = 123) | 138,236 (752,542) | 3,911–272,560 |  |
| *Pv* LDH | Control (n = 319)^a^ | 12.5 (32.8) | 8.9–16.1 | < 0.0001 |
|  | Positive (n =128) | 183,542 (650,567) | 69,754–297,329 |  |
| Pan LDH | Control (n =196) | 22.6 (8.3) | 21.5–23.8 | < 0.0001 |
|  | Positive (n =266) | 393,596 (1.3e+006) | 235,291–551,901 |  |
| CRP | NA | NA | NA |  |
|  | NA | NA | NA |  |

^a^ For pLDH assays, samples that do not express corresponding pLDH antigens were analyzed as control.


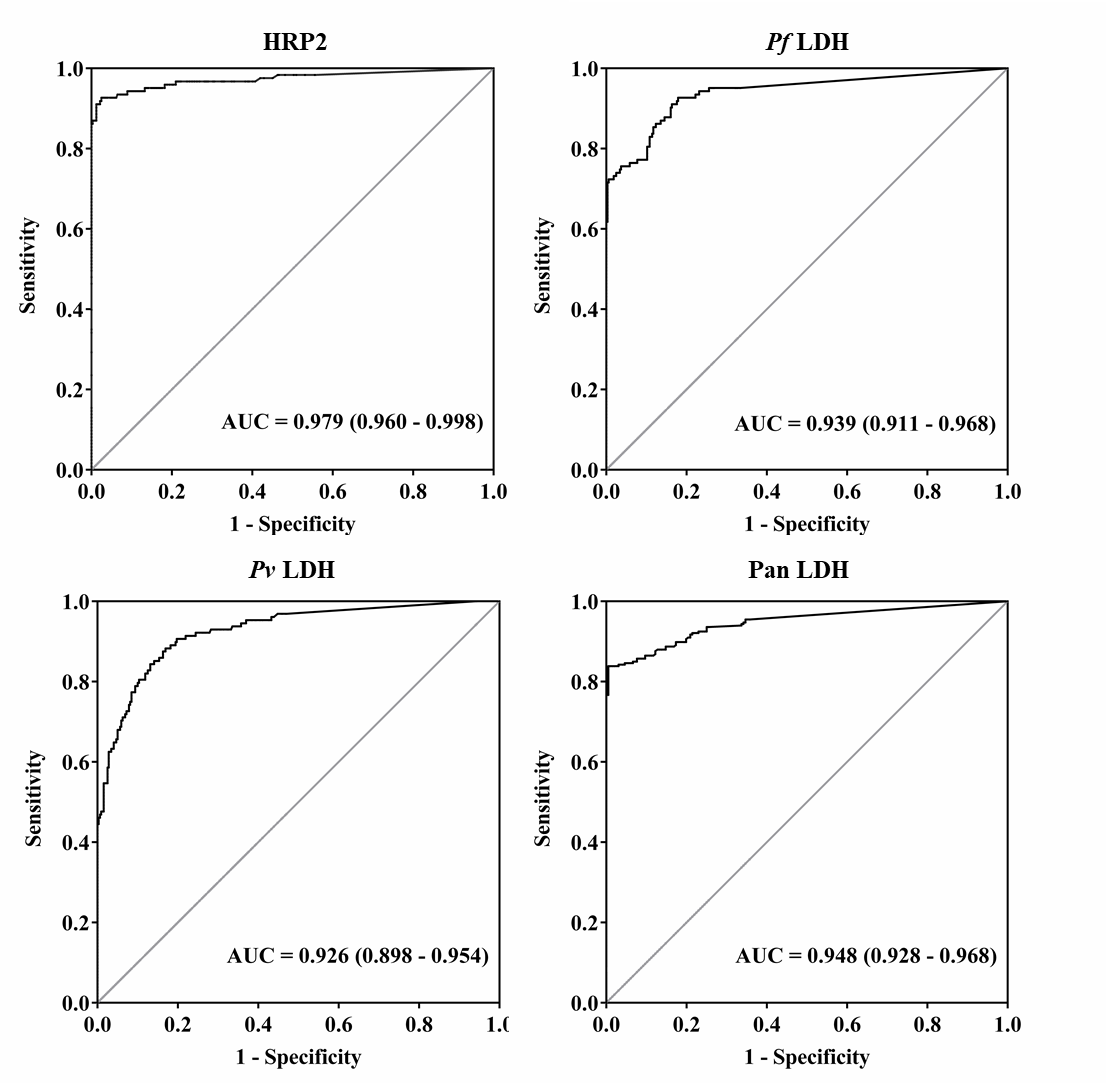


**Supplemental Figure 1.** ROC curves demonstrating the true positive rate (sensitivity) as function of false-positive rate (1-specificity) for detecting HRP2, *Pf* LDH, *Pv* LDH, and Pan LDH. Each point on the ROC curve represents a sensitivity and specificity pair resulting from applying a particular antigen concentration cutoff to determine positivity against the sample classification using positive and negative samples described in **Figure** 1. AUC (95% CI) for each curve is listed.


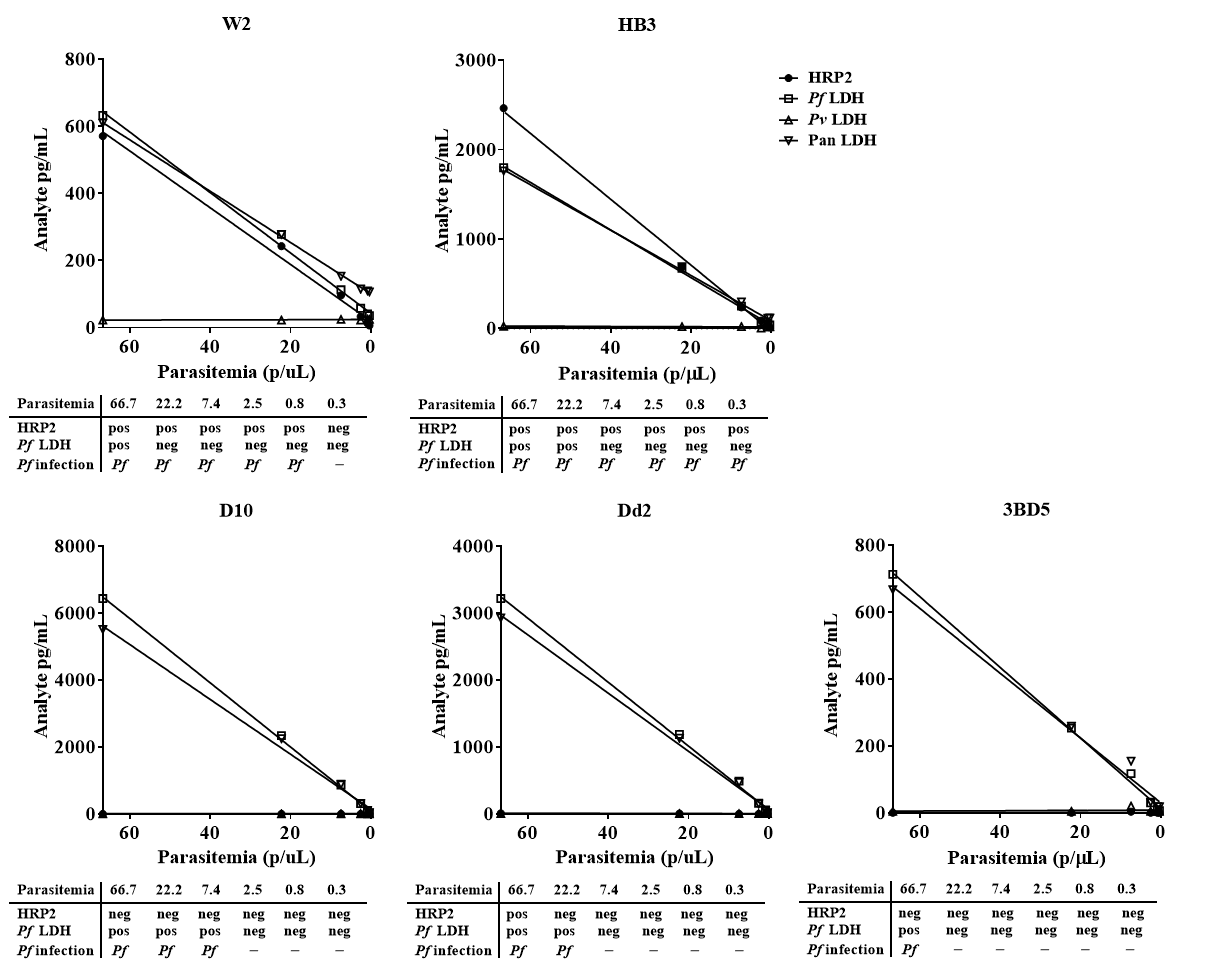


**Supplemental Figure 2.** Comparison of reactivity of malaria biomarkers in *P. falciparum* strains by the 5-Plex. Concentration of malaria biomarkers in *P. falciparum* strains, W2, HB3, Dd2, D10, and 3BD5 serially diluted in 66.7–0.3 p/μL were measured by the 5-Plex as indicated. *P. falciparum* infection status in dilution samples was determined by detection of HRP2 or *Pf* LDH above the respective cutoff values. All tested strains failed to demonstrate reactivity against *Pv* LDH assay of the 5-Plex.


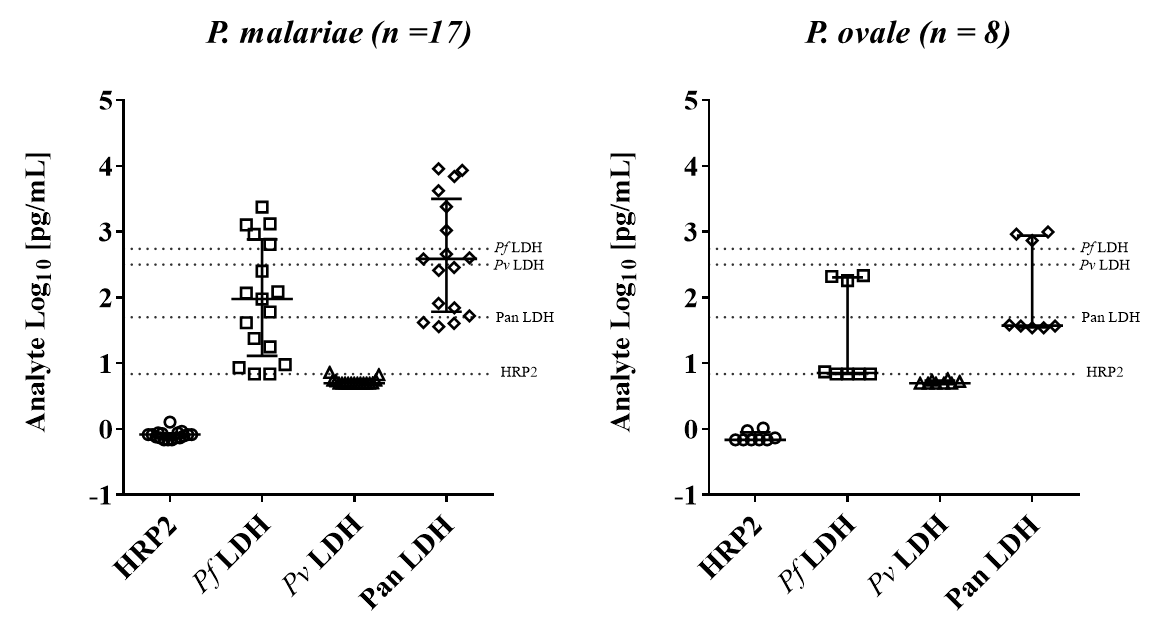


**Supplemental Figure 3.** Comparison of reactivity of pLDH from human *P. malariae* and *P. ovale* blood samples by the 5-Plex. Blood samples containing *P. malariae* (n = 17) and *P. ovale* (n = 8) were tested by the 5-Plex as indicated. The horizontal dotted lines indicate the cutoff values for HRP2, *Pv* LDH, *Pf* LDH, and Pan LDH. Five *P. malariae* blood samples with parasitemia, ranging from 75.15 p/μL to 988.3 p/μL demonstrated reactivity against both *Pf* LDH and Pan LDH assays of the 5-Plex.


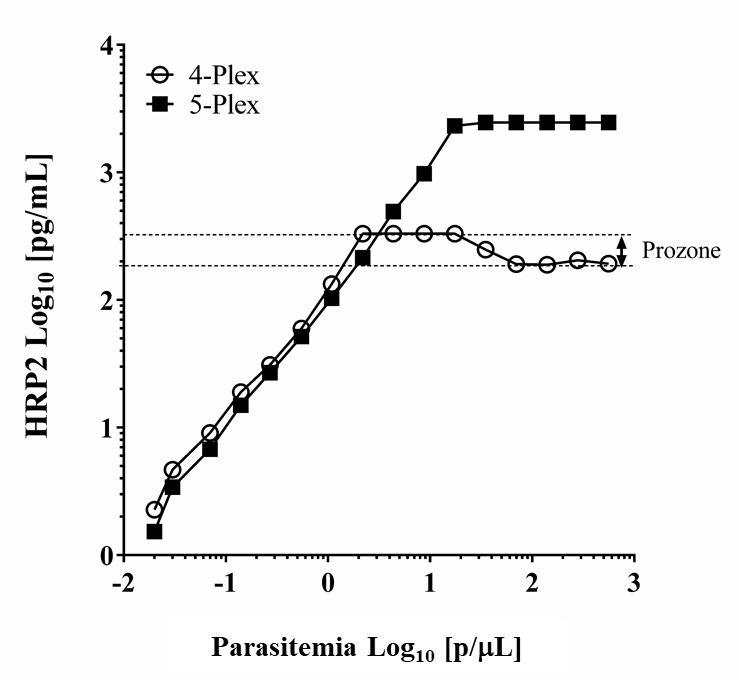


**Supplemental Figure 4.** Titration curves of the 5-Plex and 4-Plex HRP2 assays. Blood samples of *P. falciparum* in the range of 560-0.02 p/μL were tested by HRP2 assay of the 5-Plex and 4-Plex without any assay dilution. With the 4-Plex HRP2 assay, the high parasite concentrations simulate a lower parasite concentration that leads to an underestimation of HRP2 value. The 5-Plex HRP2 assay did not show a prozone effect in this range.

**Supplemental Figure 5.** Scatter plot with CRP levels from blood samples from uninfected individuals compared to those from infected individuals with or without febrile symptom. The number of blood samples in each group is indicated. Data expressed as single value for each individual with mean shown by horizontal bar; one-way ANOVA for multiple comparisons, n.s. not significant, *p* < 0.0001. CRP level was on mean of 553.9 ng/mL (median, min, and max of 156.4, 9.4, 12,265) for controls compared to mean of 1,285 ng/mL (median, min, and max of 199.3, 25.9, 35,684) for asymptomatic group and mean of 21,417 ng/mL (median, min, and max of 12,592, 1,895, 122,949) for symptomatic group.
